# Hypoxic microenvironment in cancer: molecular mechanisms and therapeutic interventions

**DOI:** 10.1038/s41392-023-01332-8

**Published:** 2023-02-17

**Authors:** Zhou Chen, Fangfang Han, Yan Du, Huaqing Shi, Wence Zhou

**Affiliations:** 1 The First Clinical Medical College, Lanzhou University, Lanzhou, Gansu China; 2 The First Hospital of Lanzhou University, Lanzhou, Gansu China; 3 The Second Clinical Medical College, Lanzhou University, Lanzhou, Gansu China; 4 Lanzhou University Sencond Hospital, Lanzhou, Gansu China

**Keywords:** Cancer metabolism, Cancer microenvironment

## Abstract

Having a hypoxic microenvironment is a common and salient feature of most solid tumors. Hypoxia has a profound effect on the biological behavior and malignant phenotype of cancer cells, mediates the effects of cancer chemotherapy, radiotherapy, and immunotherapy through complex mechanisms, and is closely associated with poor prognosis in various cancer patients. Accumulating studies have demonstrated that through normalization of the tumor vasculature, nanoparticle carriers and biocarriers can effectively increase the oxygen concentration in the tumor microenvironment, improve drug delivery and the efficacy of radiotherapy. They also increase infiltration of innate and adaptive anti-tumor immune cells to enhance the efficacy of immunotherapy. Furthermore, drugs targeting key genes associated with hypoxia, including hypoxia tracers, hypoxia-activated prodrugs, and drugs targeting hypoxia-inducible factors and downstream targets, can be used for visualization and quantitative analysis of tumor hypoxia and antitumor activity. However, the relationship between hypoxia and cancer is an area of research that requires further exploration. Here, we investigated the potential factors in the development of hypoxia in cancer, changes in signaling pathways that occur in cancer cells to adapt to hypoxic environments, the mechanisms of hypoxia-induced cancer immune tolerance, chemotherapeutic tolerance, and enhanced radiation tolerance, as well as the insights and applications of hypoxia in cancer therapy.

## Introduction

Cancer occurrence is markedly associated with increasing age, as patients aged over 60 years are more than twice as likely to develop invasive cancers than younger patients. The World Health Organization (WHO) estimates that by 2050, the proportion of the world population aged over 60 years will have increased from 12 to 22%, totaling over 2 billion individuals.^1^ In the United States, cancer is the second leading cause of death after heart disease. In humans aged over 60 years, cancer is the leading cause of death.^2^ Due to the aging of the global population, cancer has become a global public health issue, with a massive economic burden and complex treatment challenges. The distribution of oxygen partial pressure in tumor tissues is of particular interest to radiologists because the radiosensitivity of tumor tissues depends on the tissue O_2_ tension. Tumor cells living under adequate hypoxic or hypoxic conditions are relatively resistant to radiation.^3^

**Fig. 1:**
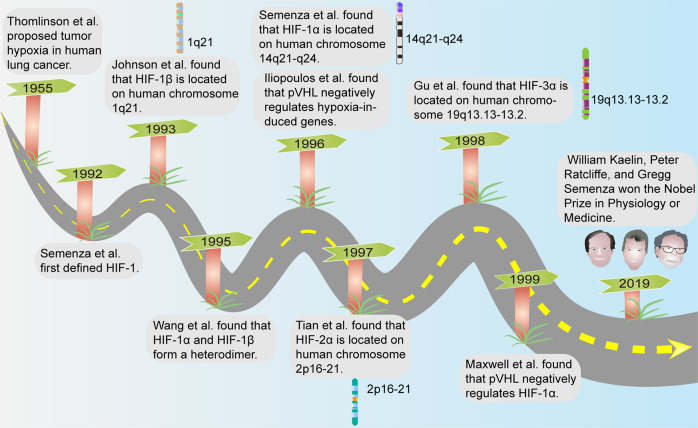
A historical and chronological figure of major events in tumor hypoxia research

In 1953, Gray et al. found that well-oxygenated tumor cells responded trice as well to radiotherapy than hypoxic cells.^4^ Tumor hypoxia was first proposed in 1955 by Thomlinson et al. in a study of tumor tissues from patients with lung cancer, and scientists have confirmed, through over 60 years of clinical and experimental evidence, that the hypoxic state is a widespread trait in a variety of solid tumors. The expression of key genes such as hypoxia-inducible factors (HIFs) and their various subunits, as well as the molecular regulatory mechanisms, were also explored under hypoxic conditions.^5–14^ William Kaelin, Peter Ratcliffe, and Gregg Semenza have been awarded the 2019 Nobel Prize in Physiology or Medicine for the contributions of several scientists who have discovered how human and animal cells sense and adapt to oxygen supply (Fig. 1).^15^ Hypoxia is present in 90% of solid tumors, which is considered a hallmark of cancer.^16,17^ It is difficult to determine the hypoxic state in tumors due to variations in oxygen content between tissues, as well as differences in tumor size and measurement methods, and tissue oxygenation is highly variable, also within the same organ.^18^ However, the available results indicate that the measurement of tumor partial pressure of oxygen (pO_2_) in patients (polarographic technique) has demonstrated the presence of low values (<10 mmHg) in several different tumor types, including pancreatic cancer, head and neck tumors, breast cancer, cervical cancer, and melanoma.^19–22^ Intra-tumor hypoxia is linked to decreased disease-free survival outcomes in several cancers including prostate, cervical cancer, and head and neck squamous cell carcinoma (HNSCC).^23–26^ The hypoxic environment alters the expression levels of genes that modulate metabolism and other processes. Moreover, hypoxic signaling interacts with other cellular pathways to alter cancer cell malignant behaviors and is closely associated with cancer cell proliferation, migration, invasion and angiogenesis, and affects cancer treatment outcomes.^27^ This article focuses on the unique hypoxic microenvironment in cancer, the possible mechanisms by which cells undergo transformation and malignancy, and the potential applications of these aspects.

## Factors contributing to hypoxia in cancer

**Fig. 2:**
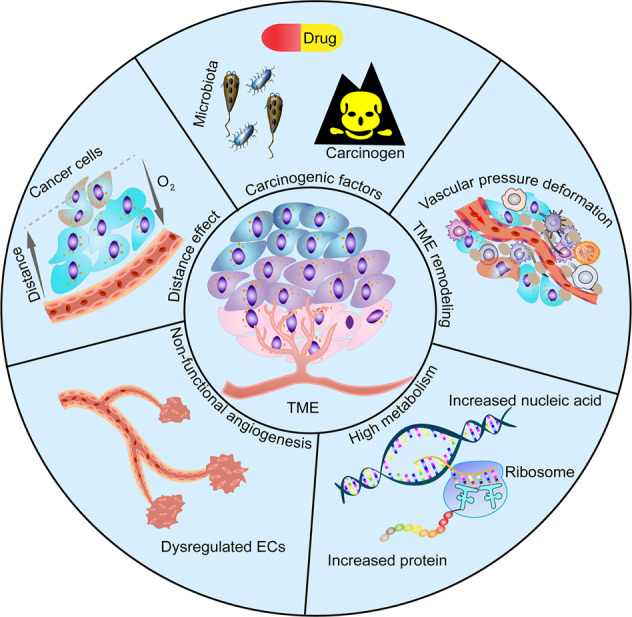
Potential factors contributing to tumor hypoxia. Carcinogenic factors, such as drug, carcinogen, and microbiota dysbiosis, impair EC shape and function in the vascular system. TME is remodeled by tumor cells, stromal cells and stromal components (e.g., fibrin), resulting in vascular deformation due to pressure. High metabolism in cancer cells, such as increased nucleic acid synthesis and increased protein anabolism, leads to relative hypoxia. Dysregulated proliferation and alignment of vascular ECs result in the formation of non-functional blood vessels. With the increased distance between tumor cells and blood vessels, O_2_ diffusion decreases and leads to hypoxia

The vasculature ensures the presence of tissue oxygen and energy substances. Vasculature endothelial cells (ECs) of cancer tissues or pre-neoplastic tissues are exposed to harmful substances, including various high-risk carcinogenic factors, such as drugs, carcinogens, pathogenic microorganisms, and the uniquely acidified tumor microenvironment (TME), which damage the shapes of ECs (for example, edema), resulting in dysregulated functions of cancer vasculature.^28^ Cancer cells are characterized by high proliferative rates and active metabolism; they also consume high amounts of energy to support their increased cell proliferation and growth rates.^29^ When metabolic oxygen demands exceed supply, the oxygen-deficient areas of cancer cells are exacerbated and they are change their metabolism.^30^ Abnormal vascular structures and patterns that are due to dysregulated angiogenesis contribute highly to hypoxia. Although the expression of erythropoietin (EPO) and angiogenic factors are increased under hypoxic conditions, which promote the proliferation of vascular ECs, the arrangement is disorganized and non-functional vessels form.^31–34^ Cancer rapidly grows such that the cancerous area is often deficient of vasculature and becomes hypoxic.^35^ In the TME, chronic hypoxia occurs as the distance of cancer cells from blood vessels increases and O_2_ diffusion decreases, which has been further validated in a mouse model of breast cancer.^36^ Hypoxia can occur in tissues more than 100–200 µM from a functional blood supply, a phenomenon that is prevalent in solid tumors.^37^ Additionally, using a model of rat brain gliom, Julien et al. found that the peritumor vasculature is vaso-responsive to hypoxia through alpha-smooth muscle actin, which can further exacerbate hypoxia in areas of tumor tissue with principle blood vessels.^38^ Some blood vessels become abnormal and malfunction, resulting in hypoxia.^39^ Non-cancer components and functions are greatly altered, including the activation and proliferation of stromal cells (for example, stellate cells and cancer-associated fibroblasts) and increased stromal components (for example, fibrin),^40,41^ leading to remodeling of cancer morphology, such as vascular compression. This can result in impaired circulation and inadequate oxygen supply, further leading to thrombosis and increased tissue hypoxia.^42^ Physiological effects of various factors, such as magnesium, change with the changing hypoxic environment. Under normoxic conditions, magnesium stimulates angiogenesis, whereas under hypoxic conditions, Mg inhibits angiogenesis.^43^ The above factors can cause hypoxia in the TME (Fig. 2).

Hypoxic factors are prevalent in most solid tumors. Some tumors contain transient hypoxic cells, others contain chronic hypoxic cells, and others contain both components. This may include three different cell populations. The first type is chronically hypoxic cells. If left in situ, the cells die. These “doomed” cells survive alone after the necessary excision in the cell survival assay and does not affect the response of the tumor when left in situ, which is considered the main cause of cell necrosis in the central region of solid tumors. The second type is the chronically hypoxic cells that are viable if left in situ. These cancer cells are stimulated by hypoxia to promote proliferation, alter gene expression, and enhance cellular drug resistance and radioresistance.^16,44–49^ The third type is transient hypoxic cells, which are expressed closer to functional blood vessels, where the duration of hypoxia is short. Based on current evidence, tumor cells adapt to hypoxia by altering their signaling pathways. Hypoxia promotes malignant behavior of cancer cells, including proliferation, migration, infestation and epithelial-mesenchymal transition (EMT), and enhances immunotherapy, chemotherapy, and radiotherapy tolerance.

## Hypoxia and carcinogenesis

### Hypoxia and genomic damage

Hypoxia damages the cellular genome and drives carcinogenesis. In in vitro and in vivo hypoxic cancer models, gene mutation frequencies of hypoxic cancer cells were increased by 2- to 5-fold.^50–52^ In tissue culture and animal models, gene amplification and mutations were associated with induction of DNA strand breaks during hypoxia, including DNA double-strand break (DSB) and single-strand break (SSB).^53–55^ In addition, hypoxic conditions (<5%) could improve the efficiency of induced pluripotent stem cell (iPSC) generation from mouse and human somatic cells.^56^ The hypoxic signaling microenvironment maintains stem cell self-renewal by facilitating the reprogramming process.^57–59^ The extrapolation of these iPSC studies provides insight into cancer stem cell (CSC), which can also exist in hypoxic ecological niches.^60^ Hypoxia is known to promote and maintain CSC phenotypes.^61^ CSCs are thought to have the potential to form tumor that will develop into cancer, especially when they metastasize with the cancer and will give rise to novel sources of cancer.^60^ Both mutation due to cellular genomic damage and the maintenance of CSCs are closely related to the production of large amounts of reactive oxygen species (ROS) under hypoxic conditions.^62,63^ The aforementioned mutations and breaks stimulate expression of oncogenes, thereby inducing the formation of cancer cell variants that have the potential to metastasize and grow.^64^

### Hypoxia and genetic repair

Hypoxia activates ataxia telangiectasia mutated (ATM) and ATM and RAD3-related (ATR) checkpoints. ATM and ATR belong to the phosphatidylinositol 3-kinase-like protein kinase (PIKK) family and are the main members of the DNA damage checkpoints. They are activated by different types of DNA damage and regulate cell cycle checkpoints by phosphorylating their corresponding downstream proteins (CHK1 and CHK2). The specificities and functionalities of ATM and ATR during DNA damage differ, with ATM being mainly involved in DNA DSB repair. After hypoxia-mediated DNA double-strand breaks, the MRE11-RAD50-NBS1 (MRN) complex activates ATM and is autophosphorylated at serine 367 (ser367), serine 1893 (ser1893), serine 1981 (ser1981), and serine 2996 (ser2996), thereby inducing MRE11-RAD50-NBS1 (MRN) complex-associated recruitment of various complex phosphorylation cascades, such as p53 (cancer suppressor), CHK1, and CHK2, to the DNA DSB sites. These effects can be blocked by the inhibition of CDK2 activity. During G1/S or G2/M cell cycle progression, cells have more time to repair DNA damage before entering mitosis. ATR kinase phosphorylates p53 and CHK1 under extreme hypoxia (oxygen concentration <0.02%).^65^ Once ATR is activated, it phosphorylates and inactivates CDC25 by phosphorylating CHK1 and CHK2, thereby resulting in failure to activate CDK2. These lesions can block G1/S or G2/M cell cycle progression by phosphorylating CDK1.^65–67^ In glioma mice models, inactivation of the ATM/CHK2/p53 pathways promoted cancer formation.^68^ Hypoxia cannot induce G2 arrest in CHK2-deficient cells, but these cells can undergo apoptosis under hypoxia.^67,69^ Persistent hypoxia enhances DNA misreplication and DNA strand breaks, resulting in mutant cell phenotypes.^70^ Non-homologous end joining (NHEJ) and homologous recombination (HR) regulate the repair of human DNA DSB.^71–74^ DNA DSB are the most severe and extensive types of damage; HR can accurately repair these damages, especially in the S/G2 cell cycle stage. NHEJ is the simplest mechanism of DNA DSB repair and can act in all cell cycle phases, except for the M phase. In hypoxic conditions, HR repairs the lesions less frequently, whereas NHEJ activities are unaffected.^75,76^ The mismatch repair (MMR) pathway can also be deregulated under hypoxic conditions. MMR is a DNA repair pathway that targets replication-related errors and primarily functions to correct the misintegration of nucleotides during DNA synthesis, thereby preventing permanent DNA damage in dividing cells. Hypoxia decreases the expression of MLH1 and MSH2, which induces mutations and dinucleotide repeat instability. These pathways may lead to sustained damage to intracellular RNA, resulting in cell transformation, including carcinogenesis.^77,78^

## Hypoxia signaling pathways and cancer cells

### HIFs and cancer cells

**Fig. 3:**
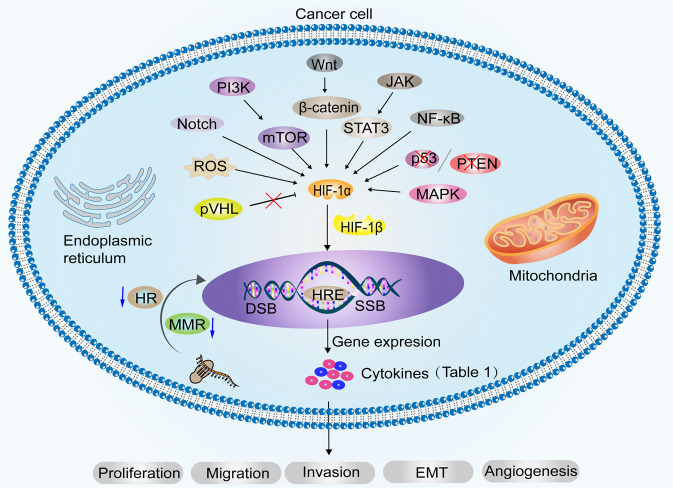
Biological changes in cancer cells adapt to hypoxia. Hypoxia promotes carcinogenesis by inducing DNA strand breaks, including DNA DSB and SSB, and by weakening DNA repair pathways, such as HR and MMR. HIF-1α is upgraded by PI3K-mTOR, JAK-STAT3, NF-κB, MAPK, Wnt/β-catenin, and Notch pathway. Deletion of tumor suppressor genes, such as p53, PTEN, and ROS production, also contributes to the upregulation of HIF-1α. The loss of pVHL function under hypoxic conditions indirectly leads to HIF-1α accumulation. HIF-1α dimerizes with HIF-1β and enters the nucleus to bind to HRE, which regulates various downstream target genes (Table 1) to promote cancer cell proliferation, migration, invasion, EMT and angiogenesis

To survive under hypoxic conditions, cancer cells reprogram their metabolism, protein synthesis, and cell cycle progression through the synergy of transcription factors.^79^ One of the main reasons why cancer cells can survive in hypoxic environments is the activation of HIFs, which reprogram metabolism, protein synthesis, and cell cycle progression.^79,80^ The HIF family has two distinct subunits: α (HIF-1α, HIF-2α, and HIF-3α) and β (HIF-1β). HIF-1α is widely expressed in all body tissues, whereas HIF-2α and HIF-3α only occur in specific tissues.^27^ HIF-1α is an oxygen-unstable protein that becomes stable in response to hypoxia, iron chelators, and divalent cations.^81,82^ In cell culture under hypoxic conditions, HIF-1α mRNA levels did not change, but HIF-1α protein levels increased.^83^ HIF-1β is constitutively expressed in mammalian cells under normoxic conditions.^84^ Owing to the presence of the oxygen-dependent proline hydroxylase family (PHD), under sufficient oxygen conditions, the HIF-α protein is hydroxylated and interacts with von Hippel-Lindau tumor suppressor protein (pVHL) to promote HIF-1α ubiquitin-proteasomal degradation.^85–87^ Ectopic expression of PHD1 inhibited HIF-1α and suppressed tumor growth.^88^ However, under hypoxic conditions, enzymatic activity of PHD is inhibited, preventing HIF-α hydroxylation and ubiquitin-mediated proteasomal degradation, leading to abnormal accumulation of HIF-α in cells. Significantly elevated mRNA of HIFs was detected after 1 h at O_2_ concentrations <10%.^89^ In addition, high expression of the CSN subunit CSN5 stabilizes HIF-1α aerobically by inhibiting HIF-1α prolyl-564 hydroxylation.^90^ Also under normoxic conditions, elevated intracellular ROS induced sustained expression of HIF-1α protein.^91^ The accumulated HIF-1α binds to HIF-1β and enters the nucleus to bind the hypoxia response element (HRE) in the promoter region of the target gene to reduce cellular oxygen consumption.^92,93^ HIF-3α exerts the opposite effects in cancer cells by impairing angiogenesis, proliferation, and metabolism.^94^ Biologically, HIF is regulated by multiple signaling pathways, including the PI3K-mTOR signaling pathway;^95–98^ JAK-STAT3 signaling pathway;^99^ NF-κB pathway;^100,101^ mitogen-activated protein kinase (MAPK) pathway;^102,103^ Wnt/β-catenin pathway;^104^ Notch pathway;^105^ cancer suppressor gene deletion, such as p53,^106^ phosphatase, and tensin homolog (PTEN);^107^ and IDH1-R132H-FAT1-ROS-HIF-1α signaling pathway (Fig. 3).^108^ Mechanistically, HIF regulates cancer cell growth by regulating genes encoding enzymes that hydrolyze sugars, angiogenic signaling genes, and apoptosis/stress response genes.^109^

### Hypoxia and cancer cell behaviors

Among various cancers, patients whose primary cancers are hypoxic at diagnosis are more likely to have local recurrence and metastatic site recurrence, regardless of whether the initial treatment is surgery or radiation therapy. Cancer patients with conditions such as anemia or chronic obstructive pulmonary disease tend to have poorer prognostic outcomes and may have cancer-related hypoxia, at least in part, owing to increased propensity to develop metastatic disease.^26,110^ Young et al. reported that exposure of cancer cells to a hypoxic environment in vitro or in vivo enhanced spontaneous metastasis.^111,112^ Similar results were obtained in mouse models of fibrosarcoma and cervical cancer.^53,113^ To an extent, hypoxic conditions eliminate tumor cells that are sensitive to hypoxia, and the surviving tumor cells adapt to hypoxia through their own molecular transformation.^114^ Several early experiments have supported that hypoxia-induced increases in HIF-1α expression can drive the metastatic phenotype by upregulating genes involved in the metastatic cascade, such as urokinase-type plasminogen activator receptor (uPAR), matrix metalloproteinase 1 (MMP1), chemokine receptor 4 (CXCR4), osteopontin (OPN), known as secreted phosphoprotein 1 (SPP1), lysine oxidase (LOX), interleukin 8 (IL-8), and vascular endothelial growth factor (VEGF).^115–123^ Hypoxia-exposed cancer cells are selective for the loss of p53 functions or increased expressions of the p53 negative regulator (MDM2), leading to increased resistance to apoptosis and increased metastasis.^124,125^ Other experiments did not find correlation between hypoxia and p53.^126^ HIF-1α binds and activates the MAX interactor-1 (MXI1), a repressor of the c-MYC transcriptional activity, and reduces the expressions of c-MYC, a factor that encodes mitochondrial DNA replication and promotes mitochondrial biogenesis,^127,128^ which inhibits cancer cell mitochondrial biogenesis and cellular oxygen consumption, resulting in cancer growth and survival in a hypoxic environment.^129^ Unfortunately, MYC overexpression occurs in 70% of human cancers.^130^ HIF-2α promotes the expressions of the MYC and E2F target genes, which are involved in lipoprotein metabolism and ribosome biosynthesis.^131^ Conversely, MYC maintains cancer stem cell self-renewal properties by selectively binding the promoter and activating the HIF-2α stemness pathway.^132^ Both HIF-1α and MYC can enhance the glycolytic pathway and drive cancer proliferation and progression.^79,133^ Under hypoxic conditions, HIF-1α directly and positively regulates ephrin A3 (EFNA3) expressions. Ephrin type-A receptor 2 (EphA2), a key functional mediator downstream of EFNA3, promotes sterol regulatory element binding protein (SREBP1) maturation, which drives the self-renewal, proliferation, and migration of hepatocellular carcinoma (HCC) cells.^35^ Under the effects of HIF-1α, prostate intraepithelial neoplasia (PIN) cells highly express transglutaminase 2 (TGM2) and exhibit impaired androgen signaling, enhancing malignant progression.^23^ In addition, some factors, such as Orai1;^134^ phospholipase D2 (PLD2);^135^ annexin A3 (ANXA3);^136^ CXCR4;^137^ cysteine-rich protein 2 (CSRP2);^138^ hematopoietic pre-B cell leukemia transcription factor-interacting protein (HPIP);^139^ twist family bHLH transcription factor 2 (TWIST2);^140^ and non-coding RNAs, such as long-stranded non-coding RNA (lncRNA) PVT1,^141^ lncRNA-GAPLINC,^142^ and LncRNA-MTA2TR;^143^ and microRNAs, such as miR-525-5p,^144^ miR-301a-3p,^145,146^ and miR-141-3p,^147^ play important roles in regulating hypoxia-induced cancer cell proliferation, migration, invasion, and angiogenesis in the TME under hypoxic conditions (Table 1).

**Table 1:** Studies related to target regulators of HIF in various cancers

Regulators	Mechanisms	Malignant behaviors	Tumors	In vitro/ in vivo	References
uPAR	HIF-1α-uPAR	Enhanced cancer cell invasion.	Lung adenocarcinoma	In vitro	^115^
MMP1	HIF-1α-MMP1	Enhanced cancer cell invasion.	Lung adenocarcinoma	In vitro	^115^
CXCR4	HIF-1α-CXCR4	Increased cancer cell migration and tumor metastasis.	Uveal melanoma	Both	^116^
OPN/SPP1	HIF-1α-CCR1- OPN	Increased cancer cell migration, invasion and metastasis.	Hepatocellular carcinoma	Both	^117^
IL-8	HIF-1α-IL-8	Increased microvascular density.	Multiple myeloma	Both	^118^
LOX	HIF-1α-LOX	Promoted tumor formation and progression.	Colorectal cancer	Both	^119^
VEGF	HIF-1α-VEGF	Promoted angiogenesis.	Hepatocellular carcinoma	Both	^120^
MXI1	HIF-1α-MXI1-MYC	Inhibited cancer cell mitochondrial biogenesis and cellular oxygen consumption, and maintains cancer cell survival.	Neuroblastoma/Renal cell carcinoma	In vitro	^127,128^
E2F	HIF-2α-E2F	Involved in lipoprotein metabolism, ribosome biosynthesis.	Clear cell renal cell carcinomas	in vivo	^131^
EFNA3	HIF-1α-EFNA3	Contributed to self-renewal, proliferation and migration in cancer cells.	Hepatocellular carcinoma	Both	^35^
TGM2	HIF-1α-E2F	Promoted cancer cell plasticity and malignant progression.	Prostate cancer	Both	^23^
Orai1	HIF-1α-Notch1-Orai1	Promoted cancer cell metastasis and angiogenesis	Colon cancer	In vitro	^134^
PLD2	HIF-1α-PLD2	Increased cancer cell migration, invasion and metastasis.	Colon cancer	Both	^135^
ANXA3	HIF-1α-ANXA3	Promoted proliferation and growth of cancer cells.	Colon cancer	Both	^136^
CXCR4	HIF-1α-CXCR4	Increased the ability of cancer cells to migrate and invade.	Gastric cancer	In vitro	^137^
CSRP2	HIF-1α-CSRP2	Facilitated hypoxia-induced cancer cell invasion.	Breast cancer	In vitro	^138^
HPIP	HIF-1α-HPIP	Promoted cancer cell proliferation, invasion, migration and EMT.	Breast cancer	In vitro	^139^
Twist2	HIF-2α-Twist2-E-cadherin	Promoted EMT.	Pancreatic cancer	In vitro	^140^
lncRNA-GAPLINC	HIF-1α-GAPLINC	Promoted cancer cell migration and invasive behavior.	Gastric cancer	Both	^142^
LncRNA-MTA2TR	HIF-1α-MTA2TR	Promoted the proliferation and invasion of cancer cells.	Pancreatic cancer	Both	^143^
miR-301a-3p	HIF-2α-miR-301a-3p	Promoted cancer cell proliferation, invasion, migration and EMT.	Pancreatic cancer	Both	^146^

## Hypoxia and cancer cell metabolism

### Hypoxia and glycolysis

The majority of cancer cells increase glucose uptake and rely on glycolysis through a phenomenon known as the “Warburg effect”.^30,148,149^ The “Warburg effect,” activated by MYC and HIF-1 in response to growth factors and hypoxia, is aerobic glycolysis with the purpose of meeting the nutrient and energy requirements for rapid genome replication. HIF-1α is the main transcription factor that promotes Warburg-like metabolism.^131,133,150^ HIF-1α stimulates various enzymes such as glycolysis regulator phosphoglycerate mutase 1 (PGAM1), pyruvate kinase M (PKM), recombinant phosphoglycerate kinase 1 (PGK1), lactate dehydrogenase A (LDHA), lactate dehydrogenase C (LDHC), and lactate dehydrogenase-5 (LDH-5) to induce anaerobic metabolic shifts that lead to energy production.^151–156^ HIF-2α promotes MYC target gene expression and enhances constitutive expression of LDHA.^131,157^ In addition, HIF-1α inactivates pyruvate dehydrogenase (PDH) by activating pyruvate dehydrogenase kinase 1 (PDK1), which in turn fails to convert pyruvate to acetyl-CoA, preventing the entry of pyruvate into the tricarboxylic acid (TCA) cycle. This leads to lactate accumulation that increases intracellular adenosine triphosphate (ATP) levels and reduces hypoxic ROS production, thus rescuing these cells from hypoxia-induced apoptosis.^158–160^

Lactate and H^+^ generated by glycolysis cross cell membranes through monocarboxylate transport proteins (that is, MCT1/4), sodium hydrogen (Na^+^/H^+^) exchanger (NHE) isoform 1 (NEH1), and carbonic anhydrase 9 (CAR9), contributing to cellular pH homeostasis and driving cancer proliferation and progression.^79,133,161–163^ Interestingly, cancer cells relying on MCT1 can autonomously consume lactate, predominating as a carbon source for the TCA cycle.^164^ Pyruvate is converted from glycolysis to lactate, while lactate is used as a respiratory fuel to support the energy and synthetic functions of the TCA cycle, which is significant for the proliferation of cancer cells.^164^

### Hypoxia and lipid metabolism

Lipid metabolism confers aggressiveness to malignant tumors by promoting membrane formation, energy storage, and production of signaling molecules, as well as by providing an important source of ATP production through fatty acid oxidation (FAO).^165^ Lipids are composed of triglycerides and lipoids such as phospholipids, cholesterol, and cholesteryl esters. Lipid metabolism involves lipid synthesis, storage, and degradation. Fatty acid (FA) are essential for lipid biosynthesis and are dependent on the activities of fatty acid synthase (FASN), adenosine triphosphate citrate lyase (ACLY), acetyl-CoA carboxylase (ACC), and stearoyl-CoA desaturases (SCD). Endogenous FA biogenesis constitutes an oncogenic stimulus that drives malignant tumor progression. HIF-1 significantly upregulates sterol regulatory element-binding protein (SREBP)-1, a major transcriptional regulator of the FASN gene, which in turn promotes FASN expression.^166^ Activation of FASN under hypoxic stress promotes *de novo* lipid synthesis and cell survival.^167,168^ ACLY is the main enzyme responsible for the production of acetyl-CoA in the cytosol in most tissues, and its product, acetyl-CoA, is used to provide a variety of biosynthetic pathways, including lipid synthesis and cholesterol synthesis. ACLY is responsible for catalyzing the conversion of citrate and CoA to acetyl-CoA and oxaloacetate.^169^ Studies have suggested that miR-27 and miR-195 are also upregulated in hypoxia-induced cardiomyocytes, suppressing the expression of ACLY and ACC.^170,171^ The mechanism of ACLY regulation in tumor cells under hypoxic conditions is still unclear. ACC (including ACC1 and ACC2) is the rate-limiting enzyme in the FA synthesis pathway.^165^ Under hypoxic conditions, the inhibition of ACC1 and ACLY increases the level of α-ketoglutarate by decreasing the level and activity of ETV4 to prevent hypoxia-induced apoptosis.^172^ ACC2 is hydroxylated by PHD3, which inhibits FAO. Under hypoxic conditions, PHD3 loss reduces ACC2 hydroxylation and promotes FAO to provide energy.^173,174^ SCD is a membrane protein of the endoplasmic reticulum that catalyzes the formation of monounsaturated FA (MUFA) from saturated FA, promotes tumor progression, and is associated with tumor recurrence and poor prognosis.^175–177^ The main products of SCD are palmitoleic and oleic acids, providing key substrates for the production of complex lipids such as triglycerides, phospholipids, and cholesterol esters. Intermittently hypoxic mouse hepatocytes upregulate SCD1 mRNA and protein by increasing SREBP-1 and serum monounsaturated FAs.^178^ HIF-1/2α promotes the progression of clear cell renal cell carcinoma by inducing SCD1 expression.^179,180^

Glutamine is the most abundant amino acid in blood and has been identified as necessary to promote mitochondrial metabolism in rapidly dividing cancer cells.^181^ HIF-1 activation leads to a significant decrease in the activity of the mitochondrial enzyme complex α-ketoglutarate dehydrogenase (αKGDH), which inhibits the oxidation of α-ketoglutarate (α-KG) as a product of the metabolic conversion of glutamine to succinate, its reductive carboxylation to isocitrate by isocitrate dehydrogenase (IDH), and then oxidation to citrate.^182,183^ HIF promotes glutamine-derived citrate conversion to cytoplasmic acetyl-CoA, which increases FA synthesis.^184^

**Fig. 4:**
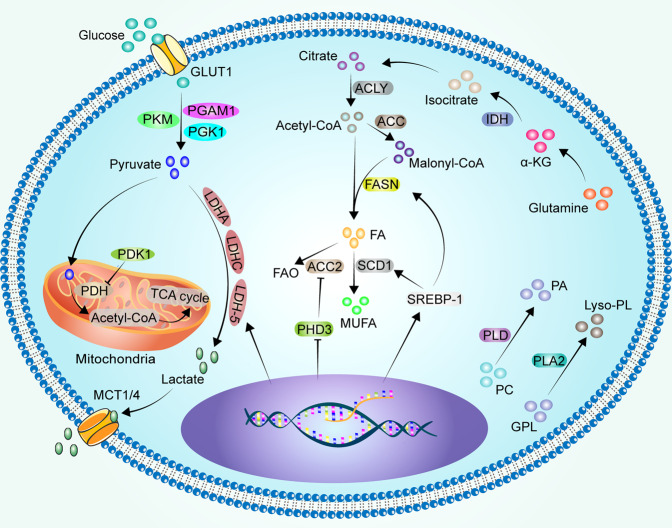
Glucose and lipid metabolism in cancer cells under hypoxic conditions. Glucose is taken up by cancer cells via GLUT1 and glycolysed to pyruvate via PKM, PGK1 and PGAM1. Hypoxic cancer cells promote pyruvate glycolysis to lactate by upregulating LDHA, LDHC and LDH-5, and the lactate produced is excreted outside the cell via MCT1/4. In addition, HIF-1α inactivates PDH by activating PDK1, which in turn fails to convert pyruvate to acetyl-CoA, preventing the entry of pyruvate into the TCA cycle. Cytoplasmic citrate is catalyzed by ALCY to acetyl-CoA, and acetyl-CoA catalyzed by ACC to malonyl-CoA. Acetyl-CoA and malonyl-CoA are catalyzed to FA via FASN upregulated by SREBP-1. SCD1 upregulated by SREBP-1 catalyzes the formation of MUFA from saturated FA. PHD3 loss reduces ACC2 hydroxylation and promotes FAO to provide energy. α-KG as a product of glutamine is reduced and carboxylated to isocitrate by IDH, and then oxidation to citrate. PLD hydrolyzes PC to produce PA. PLA2 catalyzes the hydrolysis of GPL to produce Lyso-PL

Interestingly, the lipid catabolic metabolism also contributes to cancer metastasis. Under hypoxic conditions, the main enzymes associated with lipid catabolism in tumor cells are phospholipase A2 (PLA2), phospholipase D (PLD), and carnitine palmitoyltransferase 1 (CPT1).^165^ PLA2 catalyzes the hydrolysis of glycerophospholipid (GPL) to produce lysophospholipid (Lyso-PL).^185^ Lysophosphatidic acid stimulates PLA2 phosphorylation in a HIF-1α-dependent manner, promoting ovarian cancer cell metastasis in vivo.^186^ PLD hydrolyzes phosphatidylcholine (PC) to produce phosphatidic acid (PA).^187^ Activation of the PLD1/AKT pathway increases proliferation, migration, invasion, and epithelial-mesenchymal transition (EMT) in HCC.^188^ HIF-1α up-regulates the expression of PLD2 in colon cancer cells under hypoxic conditions.^135^ In turn, PLD regulates HIF-1α at the translational level in a vHL non-dependent manner in renal cancer cells.^189,190^ Prostate cancer cells with CPT1A overexpression showed enhanced proliferation, stemness, and tumor growth compared to controls under hypoxic conditions, mildly affecting the angiogenic response.^191^ Breast cancer cells expressing CPT1C showed increased FAO, ATP production, and resistance to glucose deprivation or hypoxia.^192^ However, experimental results have shown that HIF-1α and HIF-2α inhibit CPT1A expression in clear cell renal cell carcinoma, reduce FAs transport into mitochondria, and result in FAs being stored in lipid droplets. Compared with normal tissues, CPT1A expression and activity were reduced in clear cell renal cell carcinoma patient samples.^193^ Similar results were observed in gastric cancer tissue.^194^ Cancer cells optimize their requirements for rapid growth and aggressive progression by fine-tuning the lipid anabolic/catabolic switch. However, the dominant role of this fine-tuning mechanism remains unclear. Glucose and lipid metabolism in cancer cells under hypoxic conditions is showed in Fig. 4.

## Hypoxia and angiogenesis

In the early 1970s, Folkman et al. popularized the concept of tumor angiogenesis, presenting that growing tumor cells must replenish their own blood supply to maintain oxygen and nutrients.^195^ The accumulated experimental results have shown that hypoxia favors EC proliferation and migration. Deletion of p53 in cancer cells increases HIF-1α levels and enhances transcriptional activation of HIF-1-dependent VEGF and erythropoietin (EPO) in response to hypoxia, thus promoting EC proliferation, migration, and angiogenesis.^96,106,196–199^ Hypoxia-induced E74-like ETS transcription factor 3 (ELF3) mediates increased secretions of insulin like growth factor (IGF1) and VEGF to promote EC proliferation, migration, and angiogenesis.^200^ HIF-1α mediates β-adrenergic receptor pro-angiogenesis.^39,201–203^ In addition, hypoxia stimulates the production of hyaluronic acid (a major component of the vascular basement membrane) and hyaluronidase activity, thus possibly promoting angiogenesis as a compensatory mechanism for hypoxia.^204^ However, tumor angiogenesis is not necessarily equivalent to tumor blood supply, as the discontinuous basement membrane of immature neovascularization allows for plasma and protein extravasation, further elevation of intra-tumor interstitial pressure, persistent vascular collapse, and poor nutrient delivery.^205^ In addition, some tumors exhibit an inability to maintain vascular survival, a condition that explains the well-formed, invasive peripheral and centrally necrotic hypoxic regions found in several highly angiogenic tumors.^206^ Therefore, in most cancers, despite the high vascular density, the neointima that forms are typically twisted and dysregulated, not functional for blood transport, and less efficient in oxygen and nutrient transport as well as drug delivery.^31–34,203^ Traditional anti-angiogenic strategies have attempted to reduce the vascular supply to the tumor, but their success has been limited by insufficient efficacy or the development of drug resistance. Normalization of vascular abnormalities may still be beneficial for tumor treatment with the available therapies.^31^

## Hypoxia and immune tolerance

Cancer immunotherapy has emerged as a promising strategy for the treatment of various cancers by stimulating the immune system of the patient.^207^ Programmed death receptor 1 (PD-1) and cytotoxic T lymphocyte antigen 4 (CTLA4) blocking antibodies, which are drugs approved by the Food and Drug Administration (FDA), have shown promising results in clinical trials for the treatment of various cancers. For some cancers, including breast, pancreatic, colorectal, and prostate cancers, a high proportion of non-responders has been reported, one of the main causes of which is hypoxic stress.^208^ Hypoxia-induced HIF-1α can promote programmed death ligand-1 (PD-L1) expression in cancer cells and suppress immune effects.^209^ Moreover, hypoxia plays a central role in cancer progression and resistance to therapy by promoting various changes in the biology of stromal cells in the TME, including immune cells. The main mediators of transcriptional hypoxic responses are HIF-1α and HIF-2α, which induce gene transcription, leading to hypoxic responses, and are involved in the regulation of carcinogenesis as well as stromal responses.^210^ HIF-1α has important functional roles in both innate and adaptive immune cells, including macrophages,^211^ neutrophils,^212^ dendritic cells (DCs),^213^ and lymphocytes.^214^ HIF-2α has been associated with macrophage NO homeostasis.^215^ In addition, deletion of the aryl hydrocarbon nuclear translocator (ARNT)/HIF-1β gene in CD8^+^ T cells impairs the expression of cytolytic effector molecules, including perforins and granzymes.^216^

### Hypoxia and T cells

**Fig. 5:**
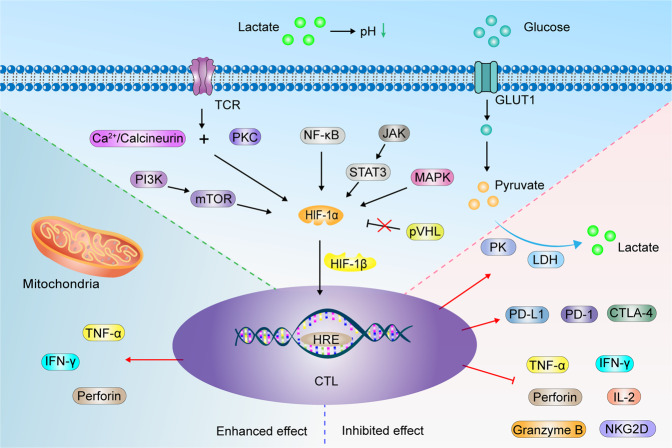
Hypoxia remodels CTL immune effect. HIF-1α in CTL is upgraded by TCR-PKC and Ca^2+^/calcineurin, PI3K/mTOR, NF-κB, NF-κB, JAK-STAT3, MAPK pathway. Deletion of pVHL impaired HIF-1α degradation. HIF-1α and HIF-1β dimerization stimulates downstream factor expression, such as perforin, IFN-γ, TNF-α, which can enhance the antitumor efficacy of CTL. However, some experiments elaborate the opposite results. Hypoxia inhibits the expression of IFN-γ, TNF-α, granzyme B, IL-2, perforin and NKG2D, while promoting PD-L1,PD-1 and CTLA-4 expression. CTL increases glucose uptake via GLUT1, which is metabolized to lactate by glycolytic enzymes PK and LDH. Lactate acidifies the TME, which inhibits CTL activation

A distinctive feature of T cells is the markedly increased glucose uptake through glucose transporter 1 (GLUT1) as they respond to immune challenges and differentiate into cytotoxic T lymphocytes (CTLs).^217,218^ Activated lymphocytes generate energy largely through upregulation of aerobic glycolysis.^217^ Glycolysis requires T cells to activate and maintain the expression of glycolytic enzymes, for example, pyruvate kinase (PK) and lactate dehydrogenase (LDH), and also requires T cells to maintain high levels of glucose uptake by maintaining the expression of glucose transporters. Relatively high levels of exogenous glucose are required to maintain the CTL transcriptional program.^219,220^ HIF-1α enhances the glycolytic activity of but not HIF-2α.^221^ Elevated HIF-α expression of T cells in hypoxic environments is regulated by multiple pathways, including the PI3K/mTOR-dependent pathway,^98^ protein kinase C (PKC) and Ca^2+^/calcineurin,^222^ STAT3 dependence,^223^ NF-κB,^100^ MAPK pathways,^103^ and T cell antigen receptors (TCRs) (Fig. 5).^98^ Deletion of VHL impairs HIF-1α and HIF-2α degradation, which significantly elevates the expression of various members of the CTL secretory granzyme family, including perforin and tumor necrosis factor α (TNF-α), thereby sustaining CTL effector functions.^224^ In natural killer (NK) cells, APOBEC3G triggered site-specific editing of C-to-U RNA under hypoxia. These effects have been shown to occur independently of HIF-1α, suggesting the existence of other potential regulatory mechanisms.^225^ HIF-α activities in CD8^+^ tumor-infiltrating lymphocytes (TILs) promote their accumulation and anticancer activities,^226^ as well as the production of interferon-gamma (IFN-γ) by CD4^+^ T cells.^227^ HIF-1α deficiency attenuates the ability of some CD8^+^ T cells to cross the endothelial barrier and reduces the abundance of infiltrating CD8^+^ T cells in the TME. This is because VEGF-A facilitates the recruitment of immune cells.^228^ VEGF-A derived from T cells promotes VE-cadherin endocytosis in ECs, causing EC adhesion junction disruption and vascular homeostasis imbalances, resulting in higher expression of the adhesion molecule (VCAM-1), which mediates immune cell adhesion to the vascular endothelium. This finding suggests that T cell-derived VEGF-A affects T cell homing through the endothelial barrier. VEGF-A levels in cancers are inversely correlated with CD8^+^ T cell levels.^221^ Loss of HIF-1α decreases the ratio of CD8^+^ to FOXP3^+^ cells in TILs.^229^ After antigenic restimulation, the generation of effector cytokines from HIF-1α mutant T cells was suppressed.^221^ Ectopic HIF-2α (but not HIF-1α) mediates the extensive changes in gene expression by altering the CD8^+^ T cell transcription factor network, regardless of VHL inhibition, including increased perforin, granzyme B, IL-2, integrins, and CXCR4. In addition, the increase in co-inhibitors such as lymphocyte activation gene-3 (LAG-3) and CTLA-4 and the decrease in IFN-γ expression is also mediated. Overall, these molecular expression changes enhance cytotoxic differentiation and lytic functions against cancer targets.^230^

However, the antitumor effects of CTLs under hypoxic conditions remain unclear. It has been reported that hypoxia impairs T cell function by reducing the levels of IFN-γ, IL-2, and NK group 2 member D (NKG2D).^231,232^ This is more in line with the actual tumor, where CD8^+^ and CD4^+^ T cells are significantly reduced in hypoxic areas of the tumor. T cell proliferations were strongly correlated with oxygen concentrations and were significantly suppressed under hypoxia (pO_2_ = 1%), likely due to hypoxia-associated changes in Ca^2+^ homeostasis in T cells.^233^ HIF-1α inhibits IFN-γ, TNF-α, and granzyme B expressions in CD8^+^, CD4^+^ T, and NK cells, which induces resistance to PD-1/PD-L1 blockade and suppresses T cell toxicity.^234^ Hyperoxia treatment suppressed the expressions of adenosine, an immunosuppressive factor,^235^ and reactive nitrogen production,^236^ and increased IFN-γ levels and perforin granules.^237^ Sustained hypoxic stimulation induces mitochondrial stress, resulting in the loss of mitochondrial functions, elevated ROS levels in T cells, T cell failure,^238^ and impeded anti-PD-1 efficacy.^239^ Because of the Warburg effect in cancer cells, which “ferments” glucose into lactic acid, lactic acid is transported outside the cell to prevent excess lactic acid accumulation. This stabilizes the intracellular pH of cancer cells while acidifying the extracellular environment.^30,133^ HIF-1α induces high expression of tumor-associated carbonic anhydrase (CA), such as CA9 and CA12, which catalyze the reversible hydration of CO_2_ to carbonic acid.^240^ In hypoxic environments, the pH of the extracellular environment of cancer cells can be as low as 5.8–6.5,^208^ which inhibits CTL activation, proliferation, and cytokine production, and also triggers T cell apoptosis.^241^

Hypoxia induces cancer cells to express chemokines such as CC chemokine ligand 28 (CCL28) to recruit regulatory T cells (Tregs).^229^ HIF-1α promotes Treg polarization and significantly contributes to colorectal cancer development and progression. This mechanism had no significant effect on the inhibition of Foxp3 expressions. Inhibiting Treg HIF-1α expression suppressed cancer growth.^242^ However, the HIF-1α-dependent transcriptional program contributes to helper T cell (Th) 17 development by mediating glycolytic activities. Moreover, HIF-1α can activate RORvt and form a tertiary complex with retinoic acid-related orphan receptor γt (ROR-γt), as well as p300, and recruit it to the IL17 promoter. In addition, HIF-1α attenuates Treg development and differentiation by binding to Foxp3 and targeting it for proteasomal degradation.^223,243,244^ Tregs express immunosuppressive factors,^245^ immunosuppressive adenosine,^246^ and cytokines such as IL-10,^247^ IL-17,^248^ and IL-35^247^ to inhibit effector T cell toxicity; therefore, they are closely associated with poor cancer prognosis.^249^ In addition, Tregs inhibit the expressions of nutrient transporters on CD8^+^ T cells, limiting nutrient uptake by T cells during activation, thereby suppressing T cell proliferation.^250^ Respiratory hyperoxia therapy may reduce the immunosuppressive effects of Tregs in the TME.^251^ Amphiregulin (Areg) is an epidermal growth factor receptor (EGFR) ligand, and Areg-EGFR promotes HIF-1α levels, leading to cancer progression.^252^ The lack of HIF-1α decreases the ratio of IFN-γ+ to FOXP3+ cells in CD4^+^ TILs, as HIF-1α deficiency reduces FOXP3 proteasomal degradation and cannot efficiently bind the IFN-γ promoter to drive Th1 responses.^227^

### Hypoxia and B cells

The differentiation process of mammalian B cells includes pro-B, pre-B, immature B, and mature B cells. HIF-1α activity, which is regulated by miR-582, is high in human and murine bone marrow pro-B and pre-B cells, promoting cell proliferation, differentiation, apoptosis, and gene rearrangement.^253^ However, reduced immature B cell stages and sustained high activities of HIF-1α reduced the abundance of surface B cell receptors (BCR), CD19, and B cell activator receptors and increased the expression of the pro-apoptotic factor (BIM), preventing normal B cell development.^254^ Although hypoxia impairs B cell proliferation, it simultaneously alters B cell metabolism and promotes antigen-mediated differentiation.^255^ HIF transcription factors suppress the activities of prolyl hydroxydioxygenase, which stabilizes HIF by hydroxylating HIF-1α and HIF-2α in conjunction with pVHL to disrupt HIF. Therefore, reducing antigen-specific B cells in germinal centers (GCs; produce long-lived plasma cells and memory B cells) disrupts high-affinity IgG production and shifts to IgG2c, early memory B cells, and recall antibody responses. In contrast, sustained hypoxia or HIF induction induced by VHL deficiency inhibits mTOR complex 1 (mTORC1) activity in B lymphoblastoid cells, which impairs B cell clonal expansion, activation-induced cytosine deaminase (AID) expression, and the ability to produce IgG2c and high-affinity antibodies.^256^ HIF-1α is closely correlated with B-cell lymphoma (BCL-6). Upregulated BCL-6 promotes the immortalization of mouse embryonic fibroblasts and primary B cells by elevating cyclin D1 levels.^257^ HIF-1α binds CXC chemokine receptor type 4 (CXCR4) to promote B-cell viability.^258^ The immune effects of B cells on cancers have not been conclusively established. Large numbers of CD20+ B cell follicles and Foxp3+ low-infiltrating cells are associated with cancer survival and better recurrence-free survival outcomes in cancer patients, including gastric and pancreatic cancers.^259,260^ Activation of HIF in B cells modulates immune responses by inducing VEGF to increase lymphatic and endothelial vessel formation and enhance DC maturation and antigen presentation.^261^ Cancer-promoting effects of B cells have been reported.^262^ Hypoxia activates HIF-1α and induces autocrine transforming growth factor-beta (TGF-β) signaling, promoting myofibroblast activation, CXCL13 induction, B lymphocyte recruitment,^263^ and factor MYC secretion, which favors B cell proliferation and survival,^264^ and drives cancer recurrence.^265^ HIF-1α-dependent glycolysis facilitates CD1d^hi^CD5^+^ B cell expansion and promotes IL-10 expressions.^266^ B cell-derived IL-35 is associated with attenuated macrophage as well as inflammatory T cell viabilities and inhibited functions of B cells as antigen presenting cells (APCs).^267^ IL-10 and IL-35 promoted cancer cell metastasis,^268^ and inhibited anticancer immune responses in mice.^269–271^ In addition, one of the mechanisms of origin of extracellular immunosuppressive adenosine relies on CD73+ and CD19+ extracellular vesicles (EVs) from B, which is regulated by HIF-1α.^272,273^ Regulatory B cells (Bregs) are a functional B cell subpopulation with the key function of secreting IL-10, thereby preventing the production of cytokines.^274^ HIF-1α is a tumor suppressor in some cancers.^275^ For instance, pancreatic-specific HIF-1α deficiency significantly accelerated Kras (G12D)-driven pancreatic adenoma formation with a significant increase in intrapancreatic B lymphocytes.^276^ Depletion of B cells increases exocrine tissue regeneration owing to a significant decrease in overall immune infiltrations and fibrosis.^277^ Pancreatitis-induced tissue hypoxia and HIF-1α accumulation-induced B-cell depletion enhances pancreatic regeneration. B cell depletion in mice with pancreatitis significantly enhanced tissue regeneration and chemotherapeutic drug resistance.^278^

### Hypoxia and macrophages

Hypoxia, HIF-1α, and tumor secretion of multiple chemokines facilitate cancer-associated macrophage (TAM) recruitment to the TME.^279–281^ TAMs constitute a plastic and heterogeneous cell population and can account for up to 50% of certain solid tumors.^282^ TAMs promote cancer progression by creating an immunosuppressive microenvironment,^283^ and enhancing the progression and metastasis of various cancers.^284–286^ Hypoxia-induced HIF-1α promotes the adaptation of TAMs to the hypoxic environment and is associated with cancer prognosis.^287^ Lipopolysaccharides (LPS) promote HIF-1α expression by stimulating the expression of Toll-like receptors in macrophages.^288^ Hypoxia (1.5% oxygen) leads to inadequate T-cell responses by increasing macrophage pro-inflammatory responses, including TGF-β, increased platelet-derived growth factor (PDGF), phagocytosis maintenance, and reduced antigen presentation. Hypoxia enhances fibrosis by promoting pro-fibrotic cytokine responses and isolating fibroblasts in the vicinity of granulomas.^289^ The hypoxic environment leads to elevated HIF-1α levels in macrophages and promotes the expression of inducible nitric oxide synthase (iNOS), which rapidly blocks T cell proliferation through NO and subsequent peroxynitrite formation.^290–292^ IL-15Rα+ TAMs downregulate CX3CL1 expression in cancer cells through the non-transcriptional activity of HIF-1α, reducing CD8^+^ T cells and/or increasing CD4^+^ T cells to reduce the ratio of CD8^+^ T cells to CD4^+^ T cells and thus weaken anti-cancer immunity.^293^ In addition, macrophages express HIF-2α under hypoxic conditions and inactivate drug-induced chemoresistance to 5-FU through HIF-2α-mediated specific overexpression of dihydropyrimidine dehydrogenase (DPD).^294,295^ Furthermore, acidic TME affect TAMs. Lactate, converted from pyruvate in cancer cells, plays a key signaling role by inducing HIF-1α-mediated expression of vascular endothelial growth factor in other processes.^296–299^ In addition, M2-like TAM promotes cancer progression by remodeling the extracellular microenvironment.^282^

### Hypoxia and NK cells

NK cells are bone marrow-derived and account for 10–18% of peripheral blood mononuclear lymphocytes.^300^ NK cells can kill various cancer targets, including cancer cells with low expressions MHC-I.^301^ Some cancer cells secrete chemokines to recruit NK cells. For instance, HCC cells are often in hypoxic microenvironments, an environment that helps CD103+ DCs to take up and clear cancer DNA, which is enhanced by blocking cell surface protein (CD47). By secreting IL-12 and CXCL9, activated CD103+ DCs induce NK cell recruitment; upregulate the expression of granzyme B, NKG2D, IFN-γ and TNF-α; and downregulate the expression of NKG2A, which in turn enhances antitumor effects. However, under hypoxic conditions, most HCC cells highly express the CD47 protein, which is associated with poor prognostic outcomes.^302,303^ Expression of NKG2D receptors, intracellular perforin, and granzyme B in NK cells is severely impaired under hypoxic conditions, which suppresses NK cell immunotoxicity.^304^ Hypoxia promotes cancer cell metalloproteinase ADAM10 expression through HIF-1α, leading to shedding of NK cell activation ligand (MICA) from cancer cell surfaces, thereby attenuating NK cell-mediated lysis.^291^ In an inflammatory model, elevated NK cell HIF-1α levels stimulated the release of IFN-γ and granulocyte-macrophage colony-stimulating factor (GM-CSF), enhancing antimicrobial defenses while promoting M1 macrophage polarization, leading to slow angiogenesis and wound healing.^305^ NK cell counts and cytotoxic effects were similarly suppressed in a chronic hypoxia mouse model, accompanied by inhibited IFN-γ secretion by NK cells. NK cells also secrete large amounts of MMP-9, which affects blood vessel remodeling.^306^ Although the absence of NK cell HIF-1α inhibits tumor growth, it is associated with various challenges. For example, deletion of HIF-1α in NK cells suppressed the expression of the angiostatic soluble form of VEGF receptor 1 (sVEGFR1) in cancer, increasing VEGF bioavailability in the TME and leading to non-productive angiogenesis. The immature vascular phenotype promotes cancer cell intravasation and distant pulmonary metastases from melanoma in vivo.^202^ Moreover, cancer cells influence the cytotoxic effects of NK cells. Upregulated HIF in the TME increases PD-L1 expression, decreases autophagic MHC-I expression, and inhibits NK cell recognition.^103,221^ HIF-2α promotes IL-10 release by activating the STAT3 signaling pathway in HCC cells, thereby inhibiting the killing activities of NK cells, which in turn promotes HCC recurrence and metastasis.^307^ HIF-2α stimulates ITPR1 expression in hypoxic cancer cells, activates autophagy in NK cells, inhibits granzyme B activity, and impairs the killing sensitivity of NK cells.^308^ Activated autophagy in hypoxic cancer cells selectively degrades the pro-apoptotic NK-derived serine protease GZMB/granzyme B, leading to the suppression of NK-mediated target cell apoptosis.^309^

### Hypoxia and neutrophils

Neutrophils are the major players in the innate immune system. Neutrophil infiltration is uncommon in normal tissues.^310^ Neutrophils are glycolytic cells that derive little ATP from oxidative phosphorylation and require gluconeogenesis to generate intracellular glycogen stores to kill bacteria.^311^ In a hypoxic environment, neutrophils reduce glycogen recycling, leading to impaired functions,^311^ and consume extracellular proteins to promote their own central carbon metabolism to maintain their functions.^312^ Infiltrating neutrophils in the TME, known as tumor-associated neutrophils (TANs), promote cancer growth and is strongly associated with poor prognosis.^313^ For instance, neutrophil-derived ROS damages hepatocyte DNA to drive HCC development.^314^ Neutrophils can eliminate bacteria from the body, forming an immune barrier that allows cancers to start growing back;^315,316^ therefore, they are closely correlated with prognostic outcomes of cancer patients.^310,317^ Elimination of neutrophil infiltration leads to cancer regression and prolonged survival outcomes.^318^ Sequencing of pancreatic single-cell transcriptome revealed that BHLHE40 is a key regulator of neutrophil polarization toward the TAN-1 phenotype, which has pro-cancer and immunosuppressive functions. However, the associated mechanism should be investigated.^319^ Neutrophils have an extensive mitochondrial network that uses the glycolytic product (glycerol-3-phosphate) to maintain polarized mitochondria and produce ROS to regulate HIF-1α stability.^320^ Neutrophil extracellular traps (NETs) promote cancer cell colonization by enhancing migration, invasion and cancer cell stemness, leading to poor cancer prognosis. Interestingly, NET formation is strongly associated with elevated HIF-1α levels in neutrophils. Downregulation of neutrophil HIF-1α can effectively inhibit NET-mediated circulating tumor cell (CTC) metastasis and prolong the median survival of mice with breast cancer lung metastasis.^321^ However, some experiments have shown the anticancer effects of NK cells. Hypoxia favors neutrophil viability and function.^322^ Hypoxia recruits polymorphonuclear neutrophils (PMNs), which are the main effector cells against endometrial adenocarcinoma growth, and induces cancer cell detachment from the basement membrane.^323^ Upon relief of tumor hypoxia, recruitment of PMNs to the TME is significantly reduced; however, the recruited cells can efficiently kill cancer cells by releasing NADPH oxidase-associated MMP-9 and ROS.^324^

### Hypoxia and MDSCs

Myeloid-derived suppressor cells (MDSCs) can suppress immune cell activity. Hypoxia and HIF-1α drive MDSCs recruitment to the TME.^280,281^ This is because cancer cells in hypoxic areas express various cytokines, such as chemokine (C-C motif) ligand 26, G-CSF, and IL-6, to recruit MDSCs.^297,325,326^ Functionally, MDSCs enhance the evasion of cancer cells from immune surveillance and promote cancer drug resistance.^327^ Inhibiting the infiltration of MDSCs improves anti-tumor effects.^328^ In an HCC model, Chiu et al. found that hypoxia-induced HIF-1α promotes the expression of ENTPD2/CD39L1, an indicator of poor HCC prognosis, which is thought to convert extracellular ATP to 5’-AMP, preventing the differentiation of MDSCs, but maintaining their survival.^329^ In addition, under hypoxic conditions, MDSCs express multiple immunosuppressive factors. For instance, PD-L1 is secreted by hypoxia-induced HIF-1α, but not HIF-2α in MDSCs,^330^ arginase and NO are promoted by HIF-1α-induced miR-210 expression,^331,332^ surface ectonucleotidases CD39 and CD73,^333^ TGF-β1, and exosomes, such as S100A9, RAR-related orphan receptor alpha (RORA), and PTEN,^281,334,335^ promote cancer cell stemness and growth and inhibit CTL function. Hypoxia through HIF-1α dramatically alters the function of MDSCs in the TME and redirects their differentiation toward TAMs, although the macrophage subtype has not been clearly established.^331^ These effects may be attributed to the ability of MDSCs to express SIRT1,^336^ a factor that regulates the glycolytic activities of MDSCs and affects the functional differentiation of MDSCs,^337^ and some miR-29a-containing exosomes.^338^

### Hypoxia and ILCs

**Fig. 6:**
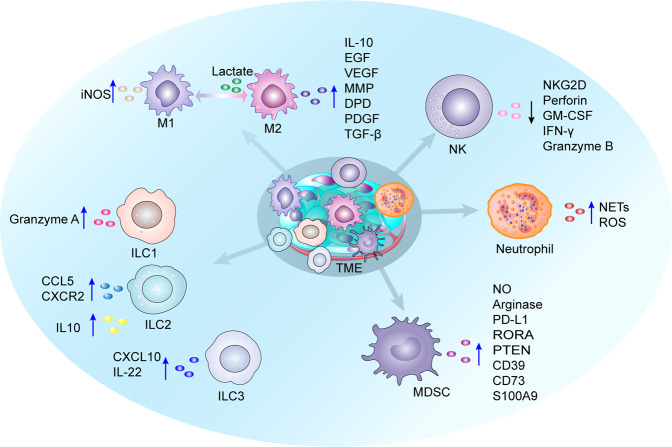
Changes in cytokine secretion by innate immune cells under hypoxic conditions. M1 microphage secretes iNOS, and M2 macrophages secrete IL-10, EGF, VEGF, MMP, DPD, PDGF and TGF-β to suppress immune responses. Lactate induces M2-like polarization. HIF-1α inhibits NK cell expression of perforin, NKG2D, GM-CSF, granzyme B and IFN-γ. Hypoxia induces neutrophils to produce ROS and NETs, which promote tumorigenesis and metastasis. HIF-1α induces MDSCs to secrete cytokines NO, arginase, PD-L1, RORA, PTEN, CD39, CD73 and S100A9, which inhibit immune response and promote the tumor cell stemness and growth. ILC1 secretes granzyme A and maintains antitumor efficacy. ILC2 enhances immune response through selective expression of CCL5, CXCR2. Hypoxia induces IL-10 expression in ILC2 and suppresses immunity. ILC3 secretes CXCL10 and IL-22, improving antitumor immune response

Innate lymphocytes (ILCs) consist of NK cells ILC1, ILC2, and ILC3, and are involved in the immune response to virus, bacteria, parasites, and transformed cells.^339,340^ Their roles in cancer immunity and immunotherapy have not been established.^341,342^ Specific changes in cancer cytokines alter ILC composition in cancers by inducing plasticity and altering ILC functions.^343,344^ IL-15 promoted ILC1 granzyme A expressions and cytotoxicity, induced the apoptosis of murine leukemia stem cells (LSC), maintained anticancer immunity, and was positively correlated with survival outcomes.^345–347^ IL33-activated ILC2 selectively expresses chemokine ligand 5 (CCL5), which recruits CD103+ DCs into the TME and activates CD8^+^ T cells to lyse cancer cells.^342^ IL-33 results in massive amplifications of ILC2 and produces CXCR2 ligands from these cells, which enhances cancer cell-specific apoptosis through CXCR2.^348^ Administration of hypoxic ILC2 resulted in a higher cancer volume, suggesting that hypoxia-exposed ILC2 enhanced the progression of pancreatic cancer cells. These outcomes were attributed to the conversion of ILC2 to ILCregs under hypoxic conditions, which inhibited T cell infiltration and IFN-γ expression by secreting IL-10.^349^ IL-25 promotes intra-tumoral ILC2 infiltration and enhancing the ability of cancer-infiltrating MDSCs to suppress antitumor immunity and reduce survival outcomes in colorectal cancer (CRC) patients.^350^ ILC3 have antitumor effects. Their intrinsic disruption in CRC drives dysfunctional adaptive immunity, cancer progression, and immunotherapeutic resistance.^351^ The production of chemokines (CCL20) and pro-inflammatory cytokines (IL-1β) at the tumor site leads to ILC3 recruitment and activation. ILC3 secrete the chemokine CXCL10, which recruits CD4^+^ and CD8^+^ T cells and promotes antitumor immune responses.^352^ IL-22, secreted by ILC3 cells, is required for initiation of DNA damage responses (DDR) after DNA damage. Stem cells that lose IL-22 signaling and are exposed to carcinogens escape DDR-controlled apoptosis, develop more mutations, and are more likely to cause colon cancer.^353^ The interaction between immune cells and TME is showed in Fig. 6.

## Hypoxia and chemotherapeutic resistance

**Fig. 7:**
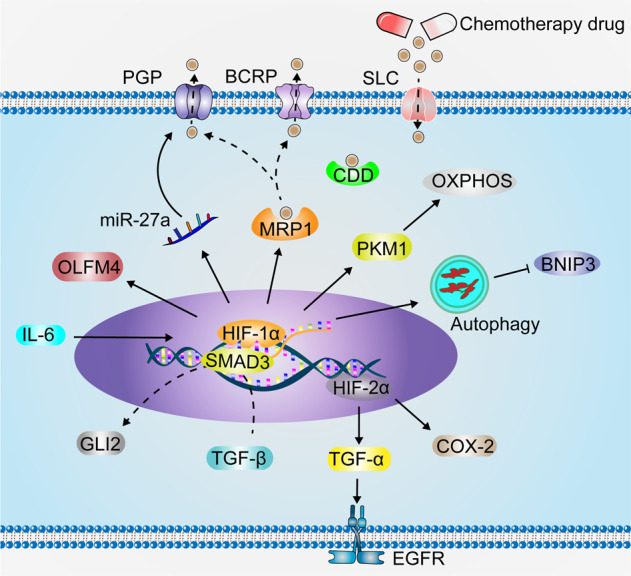
Hypoxia enhances tumor chemotherapy resistance. IL-6 stimulates HIF-1α expression, which increases PGP expression through upregulation of miR-27a. Chemotherapeutic agents are transported by HIF-1α-induced intracellular MRP1 and excreted from cells via PGP and BCRP. HIF-1α induces OLFM4 to enhance cancer cell chemoresistance. HIF-1α enhances chemotherapeutic resistance in cancer cells by promoting PKM1, enhancing mitochondrial OXPHOS. Under hypoxic conditions, cancer cells induce enhanced autophagy and inhibit the chemotherapeutic drug-induced BNIP3 death pathway to resist drug toxicity. HIF-1α synergizes with TGF-β to promote GLI2 expression through SMAD3, inducing cancer cell stemness and chemoresistance. HIF-2α upregulation promotes the ability of hypoxic cancer cells to resist drug toxicity by activating the TGF-α/EGFR pathway and COX-2. In addition, CDD and SLC play important roles in the drug resistance of cancer cells, the effect of hypoxic conditions on CDD and SLC is unclear

In vivo experiments have shown that hypoxia increases the tolerance of cancer cells to drug toxicity.^354–356^ Hypoxia induces high expression of drug resistance genes in cancer cells, increases chemotherapeutic drug efflux, and reduces intracellular drug concentrations. Once they are combined with anticancer drugs, P-glycoprotein (PGP), known as multidrug resistance protein 1 (MDR1), and breast cancer resistance protein (BCRP) are ATP-binding cassette (ABC) transporters and energy-dependent drug excretion pumps that can pump drugs out of the cell by providing energy through ATP.^357^ Therefore, the intracellular concentration of drugs continues to decrease, which weakens their cytotoxic effects until they are dissipated, and drug resistance occurs. ROS generated under hypoxic conditions induce PGP and BCRP expression through HIF-1α and increase cancer cell drug resistance.^358^ HIF-1α induces multidrug resistance-associated protein 1 (MRP1) expressions and enhances drug resistance in colon cancer cells.^359^ Moreover, HIF-1α enhances the inactivation of chemotherapeutic drugs and reduces their cytotoxic effects. Cytidine deaminase (CDD), an important metabolic enzyme involved in drug resistance development in cancer cells, is derived from cancer cells or bacteria within cancer cells. Elevated CDD levels promote the metabolism of gemcitabine to its inactive form.^360^ Moreover, solute carrier (SLC) transport proteins are primarily involved in the uptake of small molecules into cells, and their absence affects the uptake of chemotherapeutic agents into cancer cells, leading to drug resistance.^361^ The role of hypoxia in relation to CDD and SLC has not been fully established. Recently, alternative resistance pathways have been identified in hypoxic cancer cells. For instance, cancer and stromal cells in the TME, such as cancer-associated fibroblasts, secrete various cytokines, including IL-6, which induces HIF-1α expressions to regulate downstream chemotherapeutic resistance genes, such as olfactomedin 4 (OLFM4), pyruvate kinase muscle 1 (PKM1) that enhances mitochondrial oxidative phosphorylation (OXPHOS), and expressions of non-coding genes, for example, miR-27a that increases PGP expression, to promote chemoresistance acquisition in cancer cells.^362–367^ HIF-1α and cancer-associated fibroblast-secreted TGF-β signaling synergistically promote GLI family zinc finger 2 (GLI2) expressions through SMAD3, inducing CRC cell stemness and chemoresistance.^368^ Upregulated HIF-2α promotes sorafenib resistance in hypoxic HCC cells by activating the TGF-α/EGFR and COX-2 pathways.^369,370^ Under normoxic conditions, autophagy activation did not counteract cisplatin-induced stress, leading to cell death, whereas under hypoxic conditions, autophagy induction was enhanced, resolving cisplatin-induced stress and inhibiting the cisplatin-induced BCL2 interacting protein 3 (BNIP3) death pathway, allowing cell survival.^371^ Downregulation of BNIP3 may contribute to the resistance of pancreatic cancer to hypoxia-induced cell death.^372^ However, in non-small lung cancer samples, high expression of BNIP3 was significantly associated with HIF-1α and poorer overall survival was associated.^373^ Due to fibrosis and formation of non-functional vessels in the TME, drug diffusion and delivery to cells away from functional vessels is decreased^374^, and this may trigger drug resistance in cancer cells. In addition, an altered pH gradient in the TME can attenuate drug action in cells (for example, alkylating agents and antimetabolites) to enhance drug resistance.^374^ The mechanisms of hypoxia-enhanced tumor chemotherapy resistance is showed in Fig. 7.

## Hypoxia and radiation resistance

**Fig. 8:**
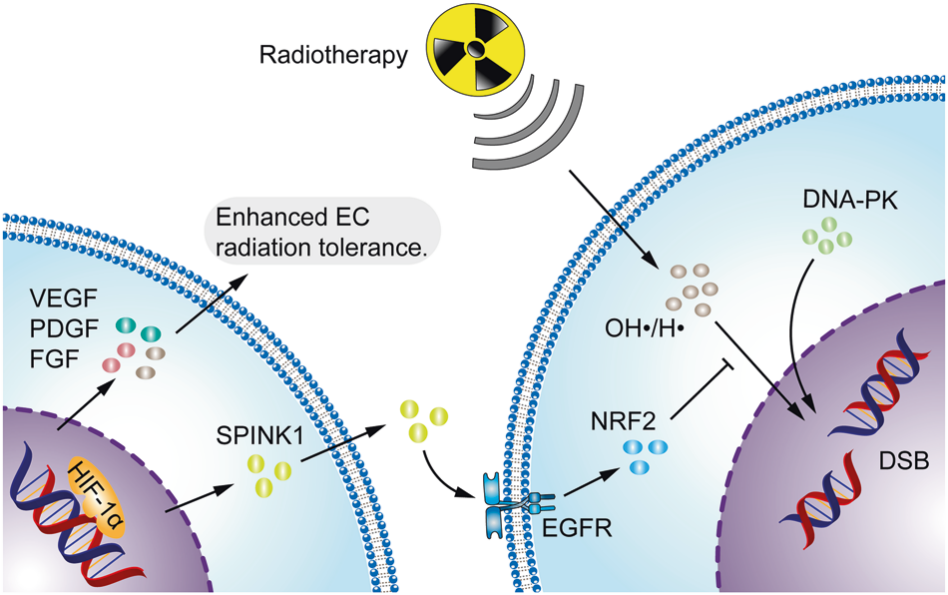
Hypoxia enhances the ability of the tumor to resist radiotherapy. Radiotherapy induces cancer cell death by directly damaging DNA through the production of free radicals such as OH• and H•. SPINK1, secreted by hypoxic cancer cells, upregulates EGFR and NRF2 antioxidant response in adjacent cancer cells to reduce radiation-induced DNA damage, thereby inducing cancer radioresistance. VEGF, FGF and PDGF secreted by hypoxic cancer cells enhanced the radiation resistance of ECs. HIF-1α stimulates DNA-PK expression and repairs DNA DSB

Radiotherapy directly destroys macromolecule fixation and leads to DNA damage through the generation of free radicals, such as hydroxyl (OH•) and hydrogen (H•) radicals, by ionizing radiation (IR), a process known as “oxygen fixation theory”. The damage caused by radiotherapy is permanent and irreversible. Hypoxia reduces the effectiveness of radiotherapy and free radicals become unstable, leading to limited extent of their damage, and most of the damage can be easily repaired.^375,376^ Classical in vitro and in vivo experiments have shown that at O_2_ (pO_2_) partial pressures below 10 mmHg, tumor cells can acquire radiobiologic hypoxia and thus become relatively resistant to radiotherapy. For instance, at 1 mm Hg, cancer cells are three times more resistant to radiation than ordinary oxygen cells.^16^ Several preclinical and clinical trials have demonstrated that the number of lethal DNA DSB formed under hypoxic conditions is also reduced by 2 to 3-fold and hypoxia can alter the expression and function of DNA DSB-associated genes. Under hypoxic conditions, additional DNA repair pathways can be activated.^16,376–379^ HIF-1α stimulates DNA-dependent protein kinase (DNA-PK) expression and repairs DNA DSB caused by ionizing radiation.^380^ Radiation upregulates HIF-1 expression and enhances its activity in cancer cells.^381^ Elevated HIF-1α levels confer radioresistance via various pro-cancer mechanisms. HIF-1α promotes ATP metabolism, and p53 activation and stimulates EC survival, thereby mediating the ultimate cancer radiation responses.^382,383^ The serine peptidase inhibitor Kazal type 1 (SPINK1) is secreted in a HIF-dependent paracrine manner to reduce radiation-induced DNA damage and enhance radiation resistance in adjacent cancer cells through EGFR and nuclear factor erythroid 2-related factor 2 (NRF2).^384^ Radiation-induced EC death leads to secondary tumor cell killing.^385^ The vascular system also affects radiation therapy. Radiation-induced secretion of VEGF, FGF, and PDGF by cancers protects the cancer vasculature from radiation-mediated cytotoxicity and enhances the radioresistance of ECs.^386–388^ Thus, VEGF, FGF, and PDGF inhibitors can significantly improve the efficiency of radiotherapy.^388,389^ The radiosensitivity of hypoxic cancer cells was showed to be significantly increased after reoxygenation.^390^ Elevated H_2_O_2_ levels in cancer tissues exacerbate hypoxia-induced resistance to radiotherapy. Reducing oncological cellular H_2_O_2_ or using it to generate oxygen through chemical reactions may improve radiotherapeutic outcomes.^391^ However, these mechanisms require further evaluation (Fig. 8).

## Hypoxia-mediated therapy

### Targeted therapy

Approximately 80–90% of deaths among cancer patients are directly or indirectly attributed to drug resistance. Progress in the development of new drugs is also hampered by drug resistance, which has become a considerable challenge in cancer treatment.^392^ Hypoxia drives cancer development and progression. Therefore, it is essential to increase the oxygen concentration in TME. Oxygen delivery in the TME is increased by enhancing cancer vascular formation to elevate oxygen levels, however, the vessels formed in cancers are usually abnormal and non-functional.^39^ Intravenous oxygen delivery to the cancer through blood is not feasible for reasons such as the potential risk of systemic ROS exposure and the low solubility of oxygen in blood.^393^ Increasing oxygen delivery by increasing blood flow to the tumor through pharmacological vasodilatation methods has been proposed, but in practice it is difficult to improve or reduce blood flow to the tumor tissue.^394^ To overcome this challenge, nano- and bio-based technologies have been used to carry oxygen generators to generate enough oxygen in the target cancer such as pancreatic ductal adenocarcinoma, colorectal cancer, fibrosarcoma, melanoma, etc., which aids in drug delivery and significantly improves the effectiveness of chemotherapy, radiotherapy as well as immunotherapy.^281,395–398^ Moreover, the reoxygenation process generates large amounts of ROS, which results in DNA damage, contributing to cancer cell death.^65,399^ However, the damage this brings to the surrounding normal cells is a matter of concern. Second, inhibition of hypoxia-induced HIF and its downstream target genes is an effective strategy.^400^ For instance, 32–134D, a low-molecular-weight compound that inhibits HIF-1/2-mediated gene expression in HCC cells, combined with anti-PD-1 increased HCC eradication rates in mice (from 25 to 67%).^401^ HIF-1α inhibitors, such as topotecan, bortezomib (PS-341) as well as HIF-2α inhibitors, such as PT2399, PT2385 and PT2977, inhibit HIF-1/2α activities and their downstream target gene expressions.^402,403^ These inhibitors reduce VEGF levels in circulating tumor cells (such as neuroblastoma, multiple myeloma, HCC and renal cell carcinoma) to inhibit tumor angiogenesis,^404–408^ and enhances the effects of chemotherapeutic drugs, such as oxaliplatin, to inhibit colorectal cancer cell proliferation.^409^ Hypoxia in the TME provides a special environment for the effects of some drugs, called hypoxia-activated prodrugs (HAPs).^395^ For instance, evofosfamide (TH-302) and tarloxotinib-effector inhibited cancer growth by suppressing signaling and cell proliferation in patient-derived cancer cells in vitro and in mouse xenograft models.^410,411^ However, there is controversy in the clinical treatment of advanced soft tissue sarcoma.^412,413^ Mechanistically, DNA dioxygenase ten-eleven translocation (TET)2 is recruited by the transcription factor (HNF4α) to activate FBP1 expressions, thereby antagonizing the functions of HIF-1/2α) in metabolic reprogramming to suppress clear cell renal cell carcinoma growth.^414^ Clinical trials regarding hypoxia-targeted therapy are summarized in Table 2.

**Table 2:** Clinical trials targeting hypoxia in tumors

Drug	Target	Cancer type	Status	Clinical trials	Study completion date	Intervention/Treatment	FDA approval
[F 18]HX4	Hypoxia regions	Head and neck cancer; Lung cancer; Hepatocellular carcinoma; Rectal cancer; Cervical cancer	Phase 2	NCT01075399	February 2012	Intravenous injection	Yes
F 18 ([^18 F] FMISO)	Hypoxia regions	Cervical adenocarcinoma; Hepatocellular Carcinoma; Glioblastoma; Brain metastases from breast cancer	Phase 2	NCT00559377 NCT02471313 NCT00902577 NCT01985971 NCT02695628	May 2014; March 7, 2018; January 31, 2018; August 19, 2016; October 31, 2018	Intravenous injection	Yes
[18 F]EF5	Hypoxia regions	Squamous cell carcinoma of the head and neck	Phase 2	NCT01774760	September 2016	Intravenous injection	Yes
Digoxin	HIF-1α	Breast cancer	Phase 2	NCT01763931	April 1, 2016	oral	Yes
Bevacizumab Tarceva Cisplatin	EGFR	Head and neck cancer	Phase 1	NCT00140556	April 2010	Intravenous infusion	Yes
Topotecan Cisplatin Bevacizumab	VEGF	Cervical cancer	Phase 2	NCT00548418	December 2012	Intravenous infusion	Yes
Erlotinib (Tarceva/ OSI-774)	EGFR	Carcinoma, non-small-cell Lung	Phase 2	NCT00983307	December 2012	Oral	Yes
BKM120/ Buparlisib	PI3K	Carcinoma, non-small-cell lung	Phase 1	NCT02128724	October 17, 2017	Oral	Yes
Avastin (Bevacizumab) Docetaxel	HIF/VEGF/VEGFR	Breast cancer	Phase 2	NCT00559754	September 2010	Intravenous infusion	Yes
Clostridium novyi-NT spores	Anaerobic bacteria	Solid tumor malignancies	Phase 1	NCT01924689	October 31, 2017	Intratumoral injection	-
TH-302	Hypoxia regions	Pancreatic adenocarcinoma; Soft tissue sarcoma	Phase 3	NCT01746979 NCT01440088	February 2016; May 2016	Intravenous infusion	Yes
Bevacizumab Irinotecan	HIF-2α/ VEGF	Glioblastoma; Medulloblastoma; Ependymoma	Phase 2	NCT00381797 NCT02076152	November 2017; April 2019	Intravenous infusion	Yes
Melatonin	HIF-1α	Oral squamous cell carcinoma	Phase 3	NCT04137627	December 2018	Oral	Yes
Everolimus (RAD001)	mTOR	Renal cell carcinoma	Phase 4	NCT01206764	July 1, 2017	Oral	Yes
Cetuximab	EGFR	Squamous cell carcinoma of the head and neck	Phase 2	NCT01104922	July 26, 2018	Intravenous infusion	Yes
Tivozanib	HIF-1/2	Renal cell carcinoma	Phase 2	NCT01297244	October 2012	Oral	Yes
Pazopanib Paclitaxel	HIF	Melanoma	Phase 2	NCT01107665	February 2018	Intravenous infusion	Yes
PT2385	HIF-2α	Glioblastoma	Phase 2	NCT03216499	June 5, 2020	Oral	Yes
Dinitrobenzamide (CB1954)	Hypoxia regions	Prostate cancer	Phase 1	NCT04374240	August 2021	Intra-prostatic injection	-
Mitomycin C	Hypoxia regions	Squamous cell carcinoma of the head and neck	-	NCT02352792	Recruiting	Intravenous infusion	Yes
Porfimycin	Hypoxia regions	Head and neck cancer	Phase 3	NCT00002507	October 2002	Intravenous injection	-
Apaziquone	Hypoxia regions	Bladder cancer	Phase 3	NCT00461591	January 2012	Intravesical	-
Tirapazamine	Hypoxia regions	Cervical cancer; Head and neck squamous cell carcinoma; Small cell lung cancer	Phase 2 Phase 3 Phase 2	NCT00003369 NCT00174837 NCT00066742	July 2004; January 2008; August 2009	Intravenous infusion	-
Nimorazole	Hypoxia regions	Head and neck squamous cell carcinoma	Phase 3	NCT01950689	January 7, 2021	Oral	-
Topotecan	HIF-1α	Small-cell lung cancer; Non-small cell lung cancer; Ovarian cancer	Phase 2 Phase 3 Phase 2	NCT00698516 NCT00390806 NCT00317772	May 2010; September 2013; November 4, 2020	Oral	Yes
Belzutifan (MK-6482/ PT2977)	HIF-2α	Clear cell renal cell carcinoma	Phase 1	NCT04846920 NCT04994522	Recruiting	Oral	Yes

These clinical trials were designed using hypoxia tracers, hypoxia-activated prodrugs, and drugs targeting HIF and downstream targets. Hypoxia tracers can visualize and quantify tumor hypoxia, assisting clinicians in the non-invasive detection and assessment of tumor hypoxia levels, and with the aid of PET imaging can be used to monitor the dynamic response of patients to treatment, potentially allowing for individualized patient treatment.^415,416^ Hypoxia-activated prodrugs can selectively target hypoxic tumor cells, have hypoxia-selective cytotoxicity, and exhibit broad antitumor activity.^412,417,418^ HIF and downstream gene-targeted drugs effectively inhibited tumor even, significantly improved patient survival, and phenocopied better tolerability.^419–421^ However, these drugs have certain toxic side effects, and common adverse events include headache, fatigue, nausea, vomiting, diarrhea and dehydration, and serious adverse events include Gastrointestinal bleeding, blood clots/deep vein thrombosis, lymphocytopenia, febrile neutropenia, anemia and triggering secondary infections. Some clinical trials do not have sufficient data to analyze the safety and efficacy of drugs.

### Hypoxia and immunotherapy

Hypoxia inhibits anti-cancer immune sensitivity.^422^ Reduction of the hypoxic environment in the TME increases the efficacy of immune checkpoint blockade (ICB) against cancers in vivo. The rational use of the pulsatile system of the TME can ameliorate hypoxia. Increasing the oxygen partial pressure through intravenous oxygenation can reverse hypoxia in the TME.^423^ However, some of the vessels in cancer are abnormal and non-functional. Therefore, vascular remodeling in the TME is necessary to ameliorate hypoxia. Elevated levels of Delta-like 1 (DLL1) in breast and lung cancer induce long-term cancer vascular normalization to alleviate cancer hypoxia, promote the accumulation of IFN-γ-expressing CD8^+^ T cells, and enhance macrophage polarization toward the M1-phenotype.^424^ Hyperoxia upregulates MHC-I expression in cancer cells, enhances T cell-mediated cytotoxicity,^398^ promotes the anti-cancer activities of NK cells,^235^ reduces the immunosuppressive effects of Tregs in the TME, and inhibits the production of MDSC-derived exosomes to reduce cancer cell stemness.^235,281^ Pro-oxidants can also be used to increase oxygen levels in the TME. Wu et al.^396^ developed nanoparticle-stabilized oxygen microcapsules that could be precisely targeted to pancreatic ductal adenocarcinoma (PDAC), improving hypoxia and significantly increasing the efficacy of anti-PD-1 antibodies against PDAC. Treatment with oxygen microcapsules combined with anti-PD-1 antibodies alleviates TAM infiltration, polarizes macrophages from the pro-cancer M2 phenotype to the anti-cancer M1 phenotype, and increases the proportion of TME Th1 cells and CTLs to enhance anti-cancer immune effects.^396^ The oxygenating agent (TH-302) eliminates hypoxia in cancers and promotes T-cell infiltration.^425^ The pro-oxidant adaptor (p66SHC) reduces ATP production in B cells by limiting glycolysis and compromising mitochondrial integrity, while activating AMPK to promote B cell mitochondrial autophagy and limiting plasma cell differentiation.^426^ Suppression of HIF pathway-mediated PD-L1 is an important measure for enhancing cancer immune responses. Inhibition of HIF-1α synthesis suppresses PD-L1 expression and induces lysosomal degradation of PD-L1, enhancing the ability of CTLs to kill cancer cells.^427^ The deletion of HIF-1α in NK cells significantly inhibits cancer growth and improves patient survival outcomes by activating the NF-κB pathway and stimulating the expression of effector molecules, such as IL-18.^428^ Single inhibition of HIF-1α in immune cells restores immune cytotoxicity; however, it also induces PD-L1 expression in cancer cells, impairing anticancer immunity.^234^ Therefore, multichannel combination therapy is vital for cancer treatment. Hypoxia leads to the increased expression of immune checkpoints and promotes fibrosis, thereby suppressing the efficacy of cancer immunotherapy. Increasing the oxygen concentration affects cancer immune responses by altering the extracellular matrix. For instance, hyperbaric oxygen (HBO) promotes PD-1 antibody delivery and T cell infiltration into the cancer parenchyma by depleting major components of the extracellular matrix, such as collagen and fibronectin.^429^ Genetic engineering approaches can be used to construct high-affinity NK (haNK) cells, which can express high-affinity CD16 receptors and IL-2, improve tolerance against acute hypoxia, and maintain the functions of NK cells to kill cancer cells.^430^

### Hypoxia and radiotherapy

Radiotherapy is a non-invasive oncologic treatment approach for drug-resistant cancers.^431^ It is based on the principle of targeting cancer tissues with X-rays or gamma rays, which directly or indirectly damage biomolecules to ionize the surrounding water and generate ROS, inducing DNA damage and apoptosis.^432,433^ Oxygen in the TME can anchor broken ends of DNA and form stable organic peroxide groups that inhibit further DNA repair.^434^ However, local hypoxia in the TME markedly reduces the therapeutic efficacy of radiation therapy. In addition, radiotherapy requires high doses of X-rays to treat cancers, which can cause serious side effects.^435^ By utilizing the sustainable production of O_2_ from endogenous H_2_O_2_ decomposition, cancer hypoxia can be improved and the efficacy of radiotherapy is enhanced.^436^ Chai et al. found that under continuous external irradiation with a 660 nm laser and X-ray beams, cyanobacteria continuously photosynthesized and released oxygen, and the large amount of ROS produced by the two-dimensional (2D) bismuth radiosensitizer greatly enhanced the efficacy of radiation therapy and inhibited cancer growth in vivo.^437^ Improving hypoxia increases the sensitivity of cancer cells to radiotherapy,^437^ which may be related to HIF-1α inhibition in cancer cells by changing the optical redox status.^438^ The mechanisms involved in hypoxia-induced tolerance to radiotherapy in cancer cells should be investigated further.

### Nano application therapy

Nanoparticles are characterized by small sizes, large surface areas, high bioavailability, and good biocompatibility, and have great potential for safely and efficiently transporting external oxygen to the hypoxic TME.^439^ The nanocomposite “oxygen bomb” PSPP-Au980-D is used to precisely locate the hypoxic microenvironment of pancreatic cancer, and through different nm laser irradiation, it can improve hypoxia, generate singlet oxygen, and enhance the efficacy of photodynamic therapy (PDT).^440^ The use of an acidic microenvironment to generate oxygen is a feasible method. For instance, an acidic environment triggers a reaction between MnO_2_ and H_2_O_2_, releasing large amounts of oxygen to alleviate intra-tumoral hypoxia. Fragmented human-induced pluripotent stem cells (iPSCs) release cancer-sharing antigens, which trigger strong innate and adaptive immune responses against cancers, promote dendritic cell maturation, and activate effector T and NK cells. Meanwhile, they also decreased the amount of Treg cells. iPS-MnO2@Ce6 significantly inhibited cancer growth, metastasis and reduced mortality in cancer-bearing mice models.^441^ Nanoparticles can also host various drugs, such as oxygenating agents, chemotherapeutic agents and immune-boosting drugs, improving the anti-cancer effects.^442^ Manganese dioxide based nanocarriers can enhance the anticancer effects of piggyback paclitaxel by alleviating the degree of hypoxia in the in situ glioma microenvironment.^442^ Porous Au@Pt core-shell nanostructures inhibit the expressions of HIF-1α and MDR1 gene by oxidizing the TME, thereby reducing the extracellular secretion of adriamycin (DOX) and enhancing the efficacy of chemo-photothermal therapy.^443^ When hybrid nanospheres containing Fe^3+^, aggregation-induced emission (AIE) photosensitizer, and Bcl-2 inhibitor of sabutoclax were consumed by cancer cells, they increased intratumoral oxygen concentration through Fe^3+^-mediated Fenton reactions, and intracellular PDT resistance of the AIE photosensitizer was mitigated by sabutoclax.^444^ Remodeling the tumor immune microenvironment by nanoparticle technology can achieve efficient cancer therapy. Mesoporous silica nanoparticles (MSNs) in combination with pro-oxidants and mitochondrial respiration inhibitors can alleviate the hypoxic environment and inhibit MDSCs infiltration.^445^ TK-M@Man-HMPB/HCQ alleviated hypoxic microenvironment-induced TAM polarization, promoted CTL infiltration, and significantly inhibited cancer growth.^446^ Novel poly (vinylpyrrolidone) (PVP)-modified BiFeO3/Bi2 WO6 (BFO/BWO) with a p-n type heterojunction reshapes the immunosuppressive TME. It triggers H_2_O_2_ catabolism to generate O_2_ to alleviate cancer hypoxia, enhance PDT and radiotherapy sensitivity, promote TAM polarization from the M2 to the M1 phenotype, and inhibit HIF-1α expression. This photo-activated nanoconjugated radiotherapy can activate and recruit T cells and stimulate TAMs toward the M1 phenotype, significantly reversing the immunosuppressive TME to immunoreactive TME and further enhancing the immune memory response.^447^ Advances in nanotechnology to improve the hypoxic TME, or to combine chemotherapy, radiotherapy, and immunotherapy, have achieved satisfactory outcomes, compared to traditional treatments, and may become a treatment measure with great potential in the future.

### Biotherapy

Accumulating evidence suggests that microbiota play an important role in cancer by damaging cellular DNA, inducing transformation, activating and altering stromal cell components in the TME, and influencing cellular metabolism.^448,449^ The microbiota characteristics under hypoxic conditions are currently not fully understood. Recent research has demonstrated that a combination of live bacteria and treatment modalities such as surgery, chemotherapy, and radiotherapy can produce good clinical outcomes. Certain bacteria have the potential to gravitate to the hypoxic core of cancer cells and can spread and proliferate. Spores of the anaerobic bacterium *Clostridium novyi-NT* were used to treat the transplanted tumors in mice. In several mouse models including colorectal cancer, biliary cancer, melanoma and squamous cell carcinoma, this bacterium was found to significantly improve the efficacy of radiotherapy.^450^ The use of the attenuated pathogenic anaerobe *Salmonella* VNP20009 to target the cancer hypoxic region, equipped with photosensitizers and bromodomain and extra terminal domain (BET) protein inhibitors with mitochondrial targeting properties improved the heat elimination abilities of photothermal therapy (PTT) and significantly inhibited the expressions of PD-L1 in cancer cells, thereby enhancing and maintaining a durable immune response.^451^ Bioluminescent bacteria designed by transforming attenuated *Salmonella typhimurium* strains effectively increased PDT and promoted inflammation by converting macrophages M2 to M1, activated NK cells, CD4^+^ Th cells and CD8^+^ T cells in the TME, reduced intratumoral immunosuppressive Tregs, and upregulated the expressions of various effector cytokines.^452^ *Salmonella* preferentially localizes sites and proliferates in hypoxic cancers, due to this advantage, *Salmonella* is also used as a cancer targeting vector to deliver different therapeutic agents and to achieve synergistic anticancer effects. However, colonized *Salmonella* recruits a large number of neutrophils, thus favoring cancer growth.^315,316^ The elimination of *Salmonella*-recruited neutrophils facilitates complete cancer eradication and reduces side effects.^453^ Delivery of peroxidase is an effective strategy for reducing resistance to radiotherapy. The peroxidase membrane vesicles (EMs) of *Escherichia coli* have higher peroxidase activity than free peroxidase, which decomposes H_2_O_2_ into oxygen to alleviate cancer hypoxia. Combined with their immunostimulatory properties, EMs can effectively enhance the effects of radiation therapy and induce anticancer immune memory.^391^ Maximizing the use of live bacteria under hypoxic conditions has the potential to be a therapeutic modality for the treatment of cancer.

## Conclusions and perspectives

Over the last 60 years, extensive progress has been made in the understanding of hypoxia-mediated signaling pathways. Hypoxia-based research has provided new insights into how hypoxia works and has laid the foundation for the development of targeted therapies that can improve patient prognosis. Carcinogenic factors and mature cancer characteristics promote hypoxia in the TME, and cancer cells adapt to hypoxia by changing their own metabolism through key genes such as HIFs. With advances in analytical tools and genomic technologies, such as pan-genomic analysis, are important for in-depth studies of HIFs.^454,455^ Hypoxia-induced pathways can alter the malignant behavior of cancer cells. Under hypoxic conditions, most downstream targets are HIF-dependent. Whether there are other key genes independent of HIFs requires further investigation. For instance, hypoxia-induced RNA editing by endogenous RNA editing enzyme can be mimicked by inhibiting mitochondrial respiration and occurs independently of HIF-1α to facilitate adaptation to hypoxic stress.^225^ Moreover, hypoxia reshapes the stromal cell properties of the TME, especially with the rise of immunotherapy, and both innate and adaptive immune cells in hypoxic environments have received extensive attention. However, despite significant breakthroughs, some results remain controversial. The reasons for this may be related to tissue specificity in addition to the experimental tools used and design bias, and mature modeling is also critical. The composition of the TME is diverse, and in addition to its own components, other foreign components may be present, such as microbiota, an emerging field that plays an important role in various cancers. Microbiota in the TME is one of the factors that form and maintain chronic hypoxia, activates HIF-1α in a non-hypoxic manner, increases HIF-1α mRNA levels, stabilizes HIF-1α protein, and induces the expression of HIF-1α regulatory genes.^288,456,457^ Changes in the characteristics of the microbiota in hypoxic environments and their effects on tumors are interesting and deserve further exploration. Therapeutic measures based on these characteristics, including targeted therapy, immunotherapy, chemotherapy, radiotherapy, radiotherapy, and biotherapy, offer good prospects. However, the complexity of the tumor relationship makes it inefficient to treat cancer from a single perspective. Multidisciplinary combination therapy, such as biology, chemistry, materials, machinery, electronics, artificial intelligence, and other multidisciplinary approaches for cancer (especially refractory cancer), is an interesting trend. Extensive study is still required before the development and implementation of efficacious and robust hypoxia-related precision treatment.
